# Supplementary material for: Sparse multi-output Gaussian processes for online medical time series prediction
Source: BMC Med Inform Decis Mak. 2020 Jul 8;20:152. doi: 10.1186/s12911-020-1069-4 (PMC7341595; doi:10.1186/s12911-020-1069-4)
Supplement: Supplementary file 1 — Additional file 1 Appendix. [file 12911_2020_1069_MOESM1_ESM.pdf]

Appendix A: Details of the hierarchical gamma prior

In this appendix, we provide more background and visualization for the hierarchical gamma prior we used for regularization. For the convenience, we use $\Gamma(\cdot)$ to denote the gamma function, and $\mathcal{G}(a, b)$ to represent a gamma distribution with shape parameter a and rate parameter b.

Following Proposition 1 in Armagan et al. (2011), for a random variable x drawn from a normal distribution with two-layered gamma priors on variance

$$x \sim \mathcal{N}(0, \psi_1), \quad \psi_1 \sim \mathcal{G}(\alpha, \delta), \quad \delta \sim \mathcal{G}(\beta, \nu), \tag{28}$$

is equivalent to the hierararchy

$$x \sim \mathcal{N}(0, 1/\rho - 1), \quad \rho \sim \mathcal{TPB}(\alpha, \beta, \nu), \tag{29}$$

where $\mathcal{TPB}(\alpha, \beta, \nu)$ denotes the three-parameter beta distribution. The probability density function of ρ is given as

$$f(\rho; \alpha, \beta, \nu) = \frac{\Gamma(\alpha+\beta)}{\Gamma(\alpha)\Gamma(\beta)} \nu^{\beta} \rho^{\beta-1} (1-\rho)^{\alpha-1} [1 + (\nu - 1)\rho]^{-(\alpha+\beta)}. \tag{30}$$

In Figure A, we visualized the density of ρ in Equation (29) for $\alpha = \beta = 0.5$, and under different values of ν (Armagan et al., 2011). In this case, the prior distribution of x is equivalent to a horseshoe prior, and ρ can be interpreted as the shrinkage coefficient (Carvalho et al., 2010).

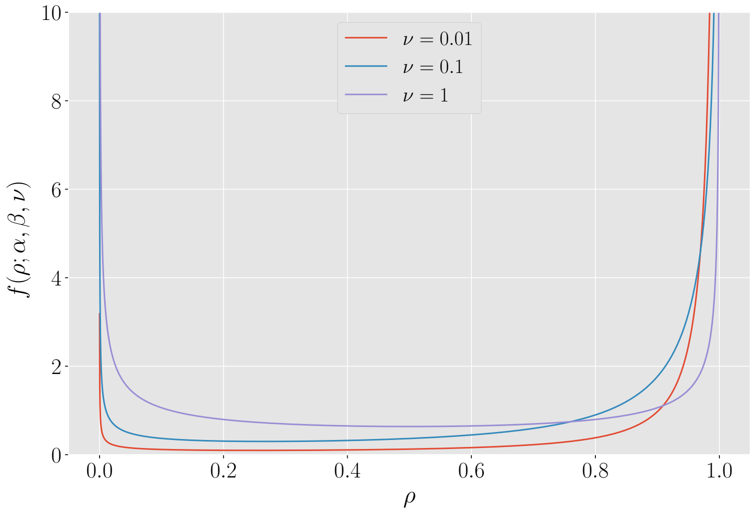

Figure A: The density of ρ drawn from a three parameter beta prior with different values of ν. For all values of ν, we set $\alpha = \beta = 0.5$.

Specifically, for the case with four layers of gamma prior used in our work,

$$x \sim \mathcal{N}(0, \psi_2), \quad \psi_2 \sim \mathcal{G}(\alpha, \delta), \quad \delta \sim \mathcal{G}(\beta, \phi), \quad \phi \sim \mathcal{G}(\gamma, \tau), \quad \tau \sim \mathcal{G}(\xi, \eta),$$

is equivalent to

$$x \sim \mathcal{N}(0, {}^1\!/\!_\rho - 1), \quad \rho \sim \mathcal{TPB}(\alpha, \beta, {}^1\!/\!_\zeta - 1), \quad \zeta \sim \mathcal{TPB}(\gamma, \xi, \eta).$$

In our case, we set $\alpha = \beta = \gamma = \xi = 0.5$ so both ρ and ζ recapitulate horseshoe priors (Armagan et al., 2011; Gao et al., 2013; Zhao et al., 2016).

References

Artin Armagan, Merlise Clyde, and David B. Dunson. Generalized beta mixtures of Gaussians. In *Advances in Neural Information Processing Systems 24*, pages 523–531. 2011.

Carlos M. Carvalho, Nicholas G. Polson, and James G. Scott. The horseshoe estimator for sparse signals. *Biometrika*, 97(2):465–480, 2010.

Chuan Gao, Christopher D. Brown, and Barbara E. Engelhardt. A latent factor model with a mixture of sparse and dense factors to model gene expression data with confounding effects. *arXiv preprint arXiv:1310.4792*, 2013.

Shiwen Zhao, Chuan Gao, Sayan Mukherjee, and Barbara E. Engelhardt. Bayesian group factor analysis with structured sparsity. *Journal of Machine Learning Research*, 17(196): 1–47, 2016.

Appendix B: Details of gradient computation and update equations

In this appendix, the equations for the objective function during optimization, update equations for the parameters in the sparsity inducing prior and the gradients for the hyper-parameters of the GP kernel are listed as reference.

The objective function to optimize for training one patient, $\mathcal{Q}(\boldsymbol{\theta})$, is

$$\begin{aligned}\mathcal{Q}(\boldsymbol{\theta}) \propto\ & \left[-\frac{1}{2}\mathbf{y}^\top (K_{|\boldsymbol{\theta}} + \boldsymbol{\epsilon} I)^{-1}\mathbf{y} - \frac{1}{2}\log|K_{|\boldsymbol{\theta}} + \epsilon I| - \left(\frac{\sum_{d=1}^{D} T_{i,d}}{2}\right)\log 2\pi\right] \\ & + \sum_{q=1}^{Q}\sum_{d=1}^{D}\sum_{r=1}^{R_q}\left(-\frac{1}{2}\log\psi_{q,(d,r)} - \frac{a_{q,(d,r)}^2}{2\psi_{q,(d,r)}}\right) \\ & + \sum_{q=1}^{Q}\sum_{d=1}^{D}\sum_{r=1}^{R_q}\left[\alpha\log\delta_{q,(d,r)} + (\alpha-1)\log\psi_{q,(d,r)} - \delta_{q,(d,r)}\psi_{q,(d,r)}\right] \\ & + \sum_{q=1}^{Q}\sum_{d=1}^{D}\sum_{r=1}^{R_q}\left[\beta\log\phi_{q,(r)} + (\beta-1)\log\delta_{q,(d,r)} - \phi_{q,(r)}\delta_{q,(d,r)}\right] \\ & + \sum_{q=1}^{Q}\sum_{r=1}^{R_q}\left[\gamma\log\tau_{q,(r)} + (\gamma-1)\log\phi_{q,(r)} - \tau_{q,(r)}\phi_{q,(r)}\right] \\ & + \sum_{q=1}^{Q}\sum_{r=1}^{R_q}\left(d\log\eta + (d-1)\log\tau_{q,(r)} - \eta\tau_{q,(r)}\right) \\ & + \sum_{q=1}^{Q}\sum_{d=1}^{D}\left(-\log 2\beta_\lambda - \frac{|\lambda_{q,(d)}|}{\beta_\lambda}\right).\end{aligned} \tag{31}$$

For update equations, we quoted from Zhao et al. (2016):

$$\hat{\psi}_{q,(d,r)} = \frac{(2\alpha-3) + \sqrt{(2\alpha-3)^2 + 8a_{q,(d,r)}^2\delta_{q,(d,r)}}}{4\delta_{q,(d,r)}} \tag{32}$$

$$\hat{\delta}_{q,(d,r)} = \frac{\alpha+\beta}{\psi_{q,(d,r)} + \phi_{q,(r)}} \tag{33}$$

$$\hat{\phi}_{q,(r)} = \frac{D\beta + \gamma - 1}{\sum_{d=1}^{D}\delta_{q,(d,r)} + \tau_{q,(r)}} \tag{34}$$

$$\hat{\tau}_{q,(r)} = \frac{\gamma + d}{\phi_{q,(r)} + \eta} \tag{35}$$

$$\frac{\partial}{\partial\theta_j}\log p(\mathbf{y}|\mathbf{x},\boldsymbol{\theta}) = \frac{1}{2}\mathrm{tr}\left(\left(\boldsymbol{\alpha}\boldsymbol{\alpha}^\top - K_{|\boldsymbol{\theta}}^{-1}\right)\frac{\partial K_{|\boldsymbol{\theta}}}{\partial\theta_j}\right) \quad \text{where } \boldsymbol{\alpha} = K_{|\boldsymbol{\theta}}^{-1}\mathbf{y}, \theta_j \in \boldsymbol{\theta} \tag{36}$$

$$
\begin{aligned}
\frac{\partial \mathcal{Q}(\boldsymbol{\theta})}{\partial a_{q,(d,r)}} &= \frac{1}{2}\mathrm{tr}\left(\left(\boldsymbol{\alpha}\boldsymbol{\alpha}^\top - K_{|\boldsymbol{\theta}}^{-1}\right)\frac{\partial K_{|\boldsymbol{\theta}}}{\partial a_{q,(d,r)}}\right) - \frac{a_{q,(d,r)}}{\psi_{q,(d,r)}}, \\
\text{where} \quad \frac{\partial K_{|\boldsymbol{\theta}}}{\partial a_{q,(d,r)}} &= B'_q \otimes k_q(\mathbf{x}, \mathbf{x}'), \\
B'_{q,(i,j)} &= \begin{cases} 2a_{q,(d,r)} & \text{, for } i = j = d, \\ a_{q,(j,r)} & \text{, for } i = d, j \neq d, \\ a_{q,(i,r)} & \text{, for } i \neq d, j = d, \\ 0 & \text{, otherwise.} \end{cases}
\end{aligned} \tag{37}
$$

For partial gradients used for optimization:

$$
\begin{aligned}
\frac{\partial \mathcal{Q}(\boldsymbol{\theta})}{\partial \lambda_{q,(d)}} &= \frac{1}{2}\mathrm{tr}\left(\left(\boldsymbol{\alpha}\boldsymbol{\alpha}^\top - K_{|\boldsymbol{\theta}}^{-1}\right)\frac{\partial K_{|\boldsymbol{\theta}}}{\partial \lambda_{q,(d)}}\right) - \frac{\mathrm{sign}(\lambda_{q,(d)})}{\beta_\lambda}, \\
\text{where} \quad \frac{\partial K_{|\boldsymbol{\theta}}}{\partial \lambda_{q,(d)}} &= \mathrm{diag}\left(\boldsymbol{\lambda}'_q\right) \otimes k_q(\mathbf{x}, \mathbf{x}'), \\
\lambda'_{q,(i)} &= \begin{cases} 1 & \text{, for } i = d, \\ 0 & \text{, otherwise.} \end{cases}
\end{aligned} \tag{38}
$$

$$
\begin{aligned}
\frac{\partial \mathcal{Q}(\boldsymbol{\theta})}{\partial v_q} &= \frac{1}{2}\mathrm{tr}\left(\left(\boldsymbol{\alpha}\boldsymbol{\alpha}^\top - K_{|\boldsymbol{\theta}}^{-1}\right)\frac{\partial K_{|\boldsymbol{\theta}}}{\partial v_q}\right), \\
\text{where} \quad \frac{\partial K_{|\boldsymbol{\theta}}}{\partial v_q} &= B_q \otimes k_{qv}(\mathbf{x}, \mathbf{x}'), \\
k_{qv}(\mathbf{x}, \mathbf{x}') &= -2\pi^2\tau^2 \exp(-2\pi^2\tau^2 v_q)\cos(2\pi\tau\mu_q).
\end{aligned} \tag{39}
$$

$$
\begin{aligned}
\frac{\partial \mathcal{Q}(\boldsymbol{\theta})}{\partial \mu_q} &= \frac{1}{2}\mathrm{tr}\left(\left(\boldsymbol{\alpha}\boldsymbol{\alpha}^\top - K_{|\boldsymbol{\theta}}^{-1}\right)\frac{\partial K_{|\boldsymbol{\theta}}}{\partial \mu_q}\right), \\
\text{where} \quad \frac{\partial K_{|\boldsymbol{\theta}}}{\partial \mu_q} &= B_q \otimes k_{q\mu}(\mathbf{x}, \mathbf{x}'), \\
k_{q\mu}(\mathbf{x}, \mathbf{x}') &= -2\pi\tau \exp\left(-2\pi^2\tau^2 v_q\right)\sin\left(2\pi\tau\mu_q\right).
\end{aligned} \tag{40}
$$

References

Shiwen Zhao, Chuan Gao, Sayan Mukherjee, and Barbara E. Engelhardt. Bayesian group factor analysis with structured sparsity. *Journal of Machine Learning Research*, 17(196): 1–47, 2016.

Appendix C: Detailed results of imputation error and 95% coverage

We organized the detailed results of online imputation on all 24 covariates under the best number of basis kernel ($Q = 5$ for HUP subsets and $Q = 4$ for the MIMIC-III subset) in Figure B to Figure E. For Figure B and Figure C, the mean absolute errors (MAEs) for each covariate is shown (in the original unit of measure). In Figure D and Figure E, we showed the percentage for the prediction lied within the 95% confidence region (i.e. 95% coverage). We put markers in the figures to indicate the best among all methods, and the comparison of MedGP (sparse SM-LMC with online updating) against other methods. The statistical significance were tested using paired t-tests on patient-level results.

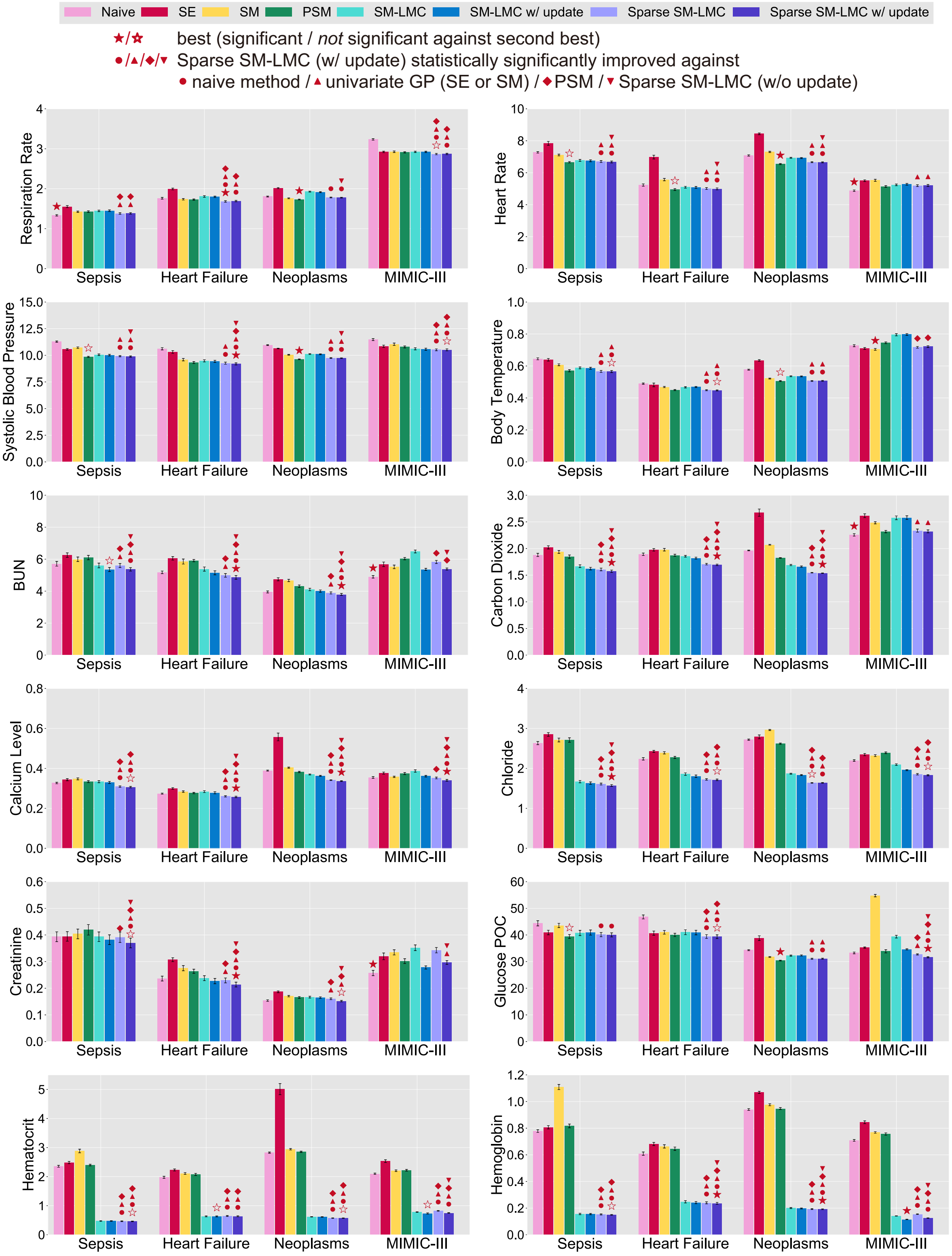

Figure B: Mean absolute error (MAE) for 12 out of 24 covariates tested. The error bars denote ±1 standard error.

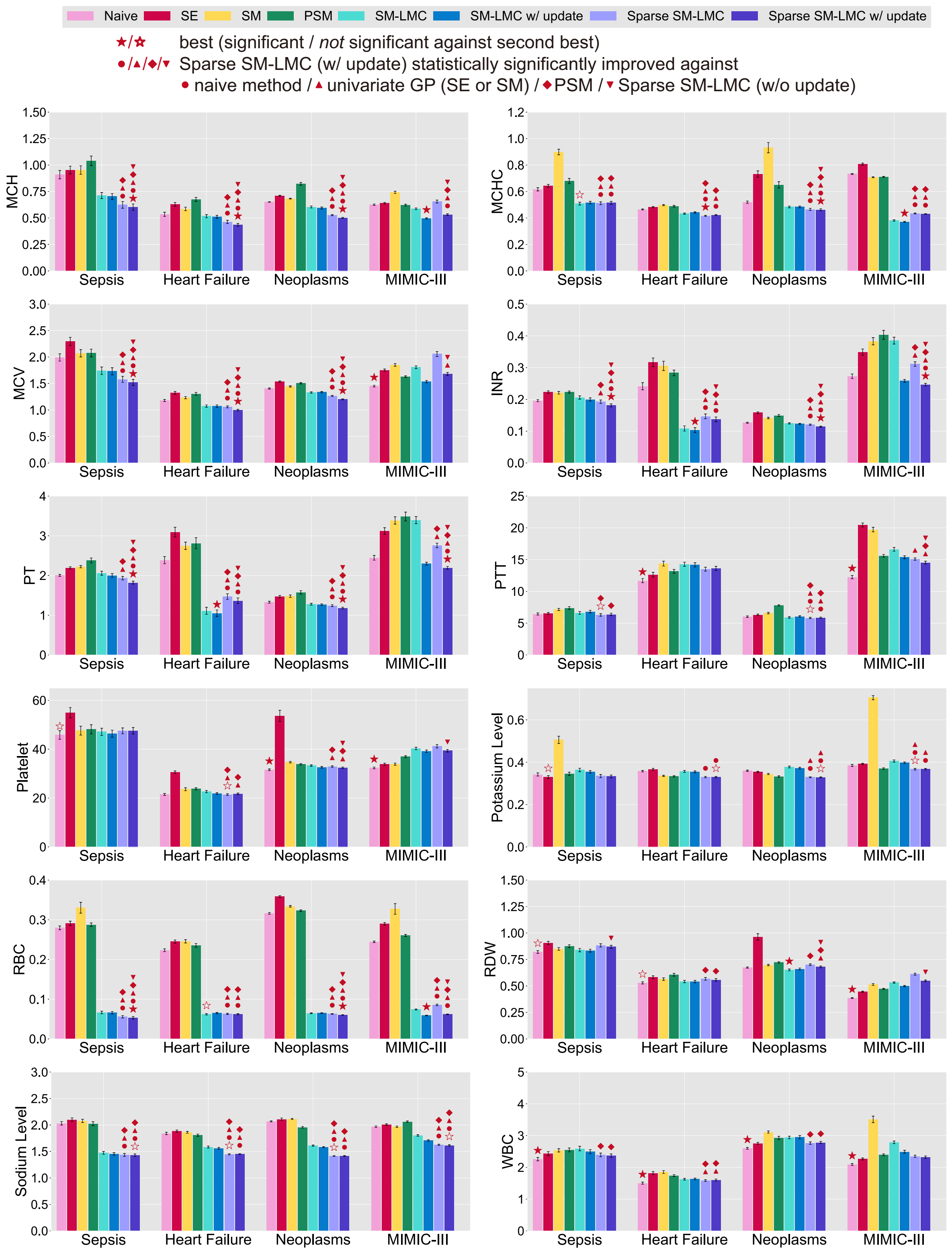

Figure C: Mean absolute error (MAE) for 12 out of 24 covariates tested. The error bars denote ±1 standard error.

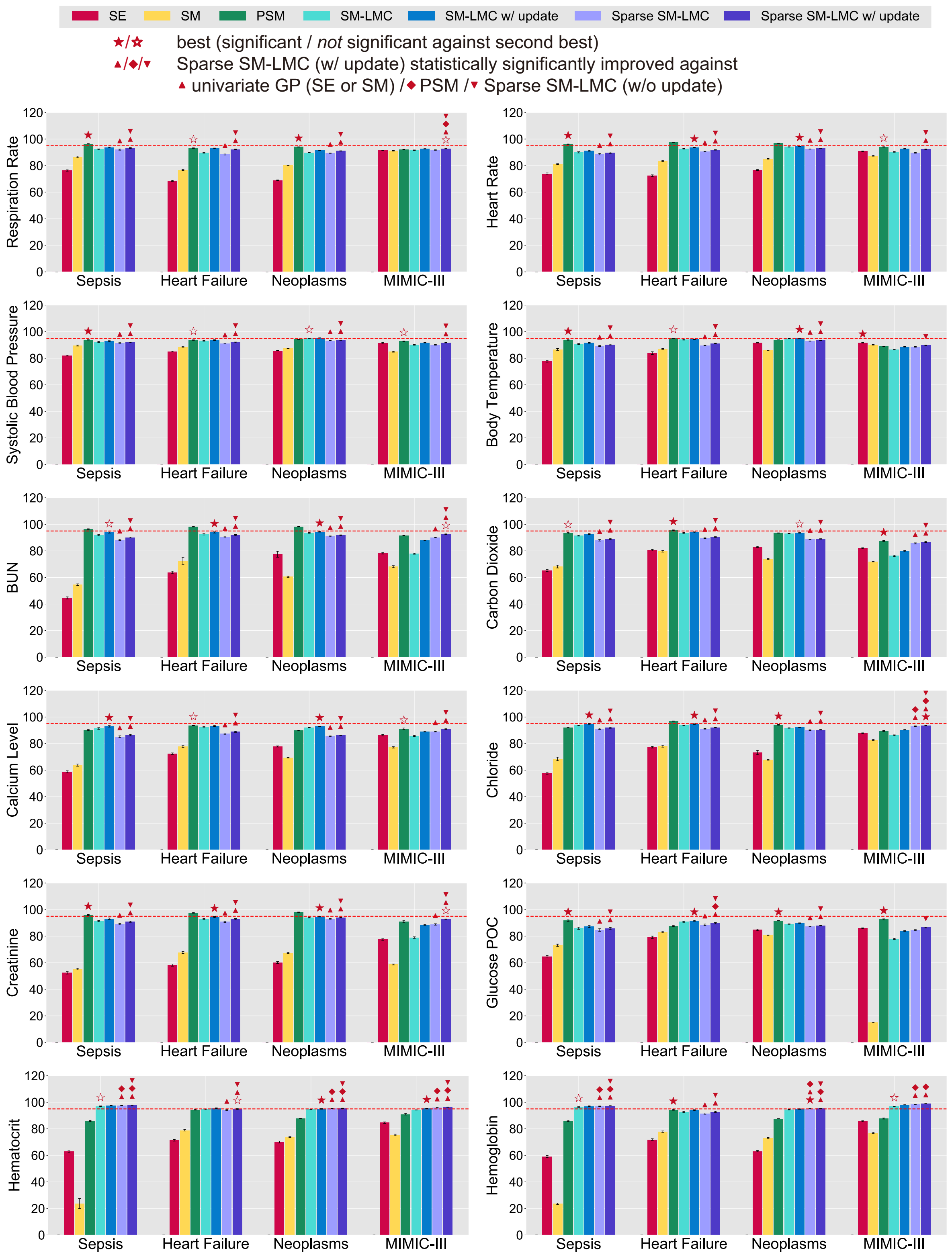

Figure D: The 95% coverage for 12 out of 24 covariates tested. The error bars denote ±1 standard error. The red dashed line indicates 95%.

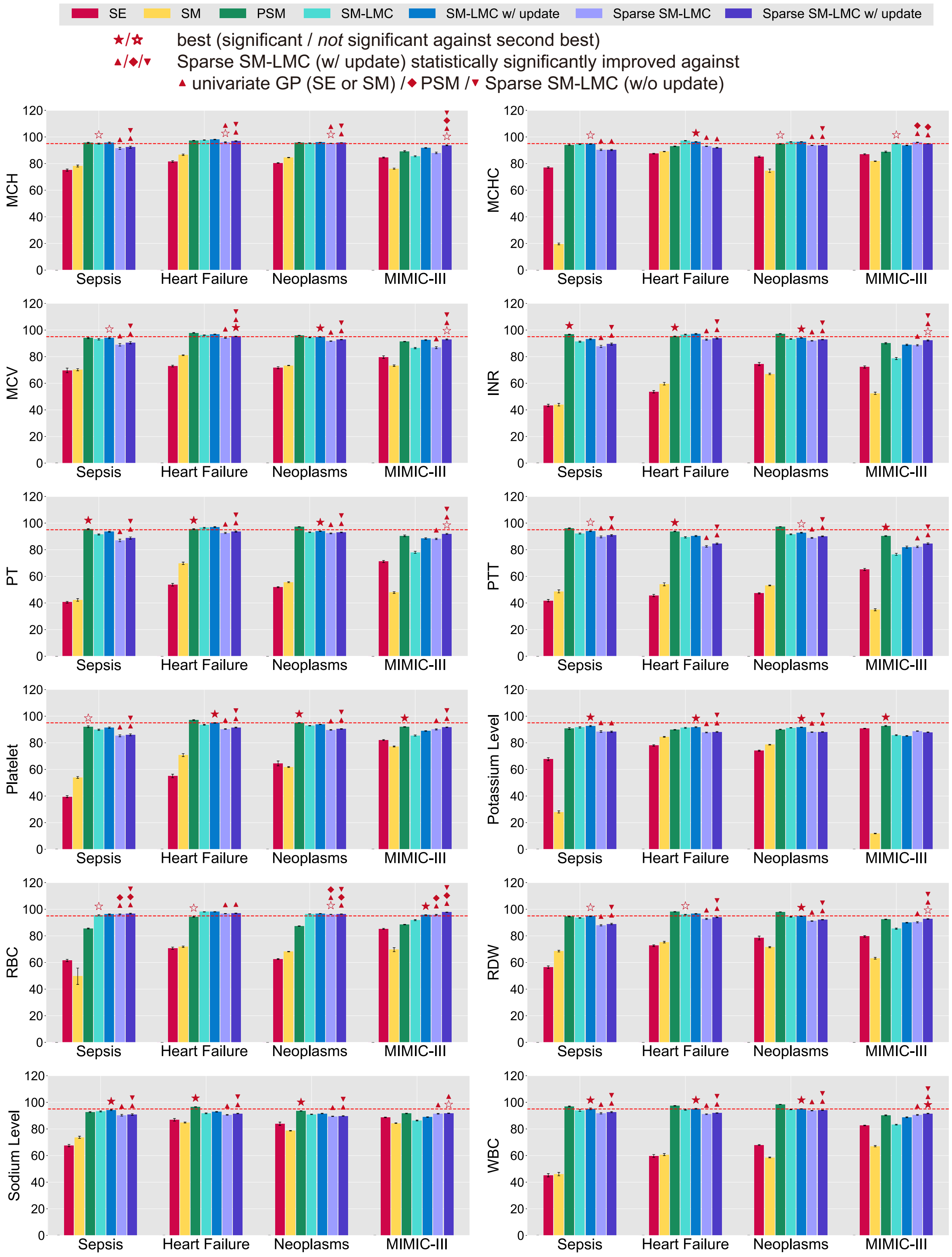

Figure E: The 95% coverage for 12 out of 24 covariates tested. The error bars denote ±1 standard error. The red dashed line indicates 95%.

Appendix D: Results under different number of basis kernels

In this appendix, we showed more detailed results of the experiments using different number of basis kernels. We ran experiments with for $Q = 1, \cdots, 5$ on all four subsets. The results include all three subgroups in the HUP data set and the MIMIC-III heart failure subset. We visualized the results in Figure F–U. We noticed that for most of the covariates, the imputation performance (both MAE and 95% coverage) improves as the number of Q increases. We also observed that the best number of Q varies across covariates under different metrics. For instance, for lab covariates INR and PT, we observed that setting $Q = 1$ or $Q = 2$ reduces MAE compared with $Q = 5$, but the coverage still improves after $Q = 2$. Allowing more numbers of basis kernels increases the flexibility for customization, but also increases complexity and thus the risk of overfitting for some covariates or patients. Overall $Q = 5$ for HUP subsets and $Q = 4$ for the MIMIC-III subset reached the largest number of covariates improved over the best of baselines using imputation error as the performance metric. How to improve the performance for a specific clinical covariate at patient-level would be one future direction of interest.

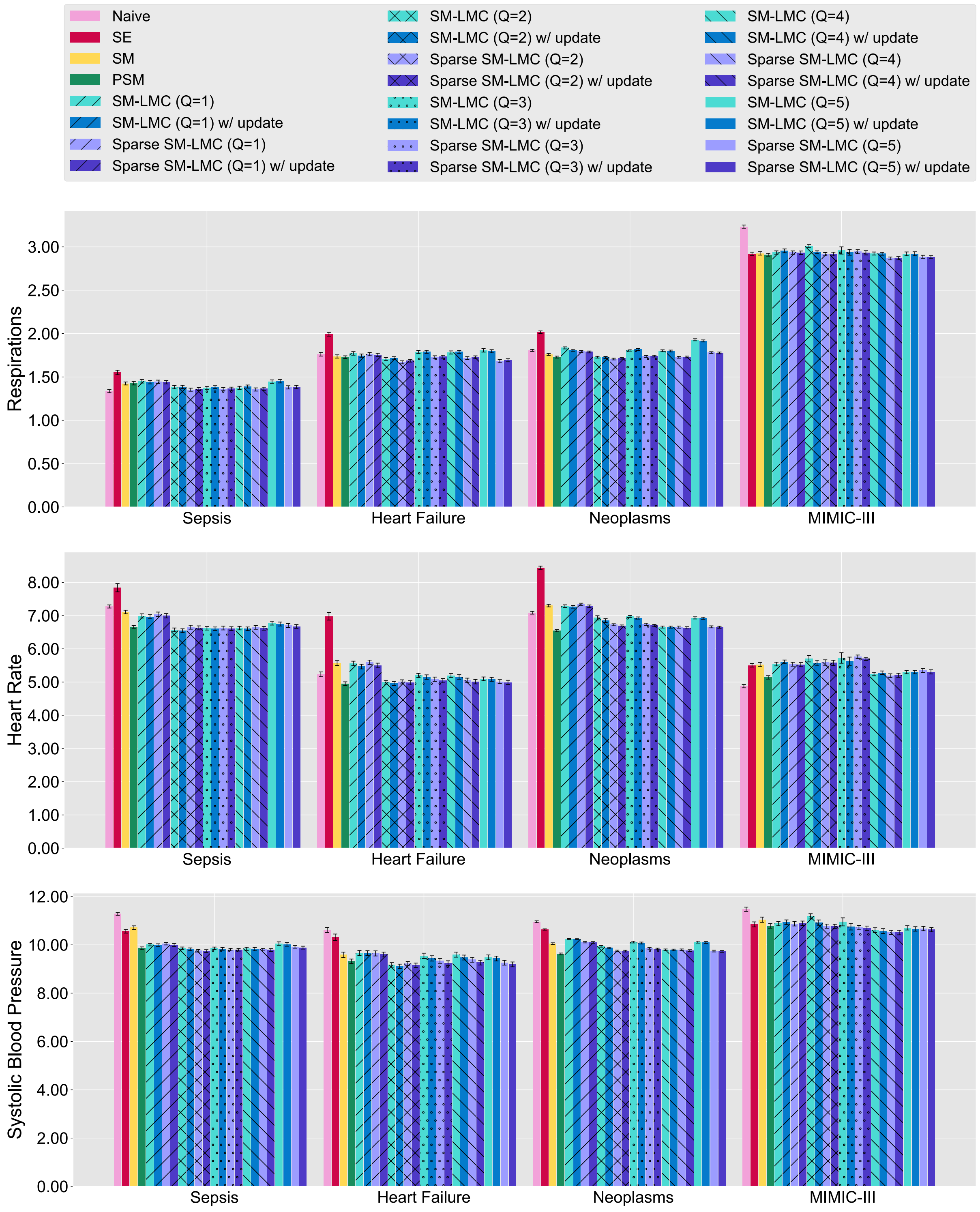

Figure F: The mean absolute error (MAE) of online imputation under different Q for all cohorts. The error bars denote ± 1 standard error.

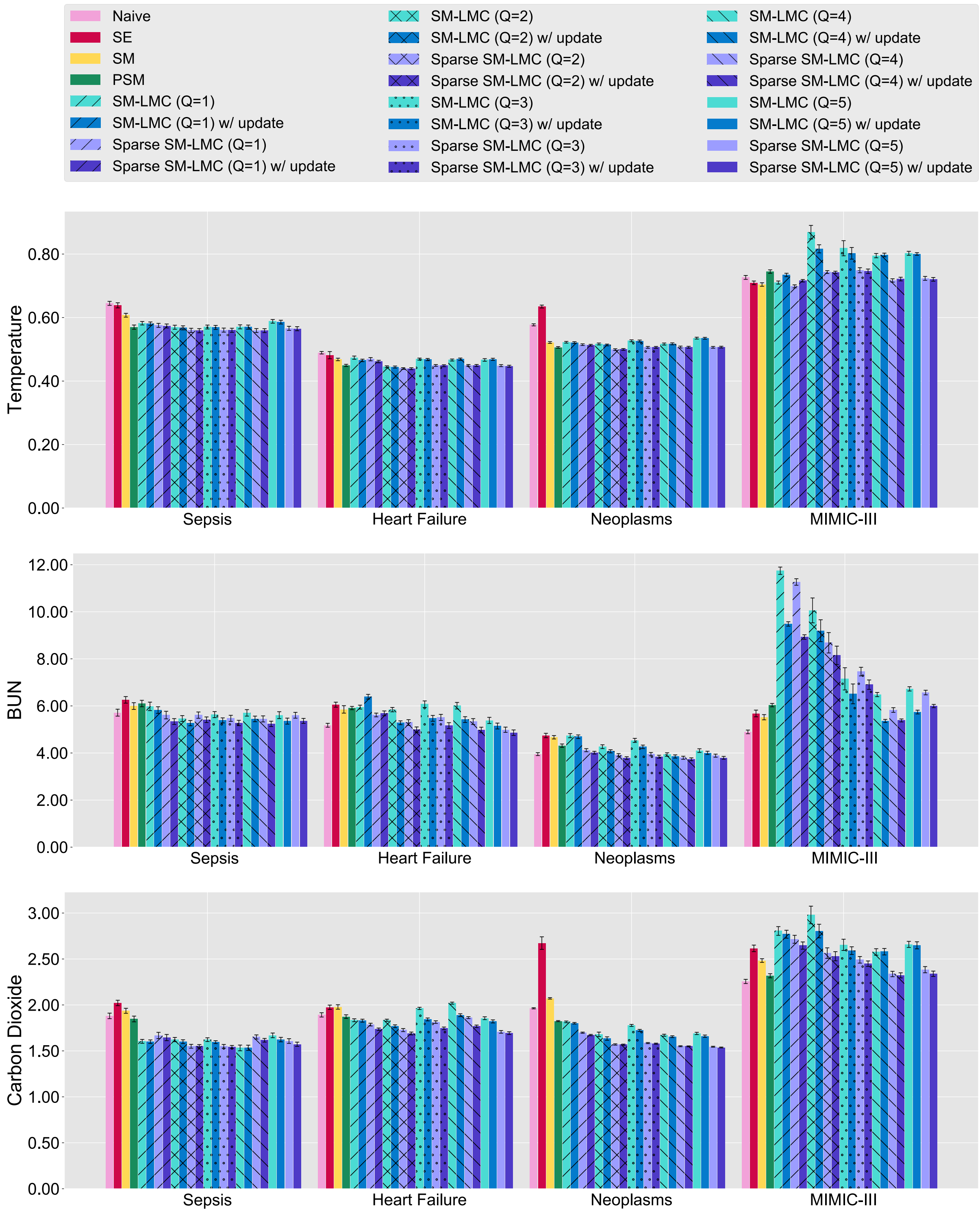

Figure G: The mean absolute error (MAE) of online imputation under different Q for all cohorts. The error bars denote ± 1 standard error.

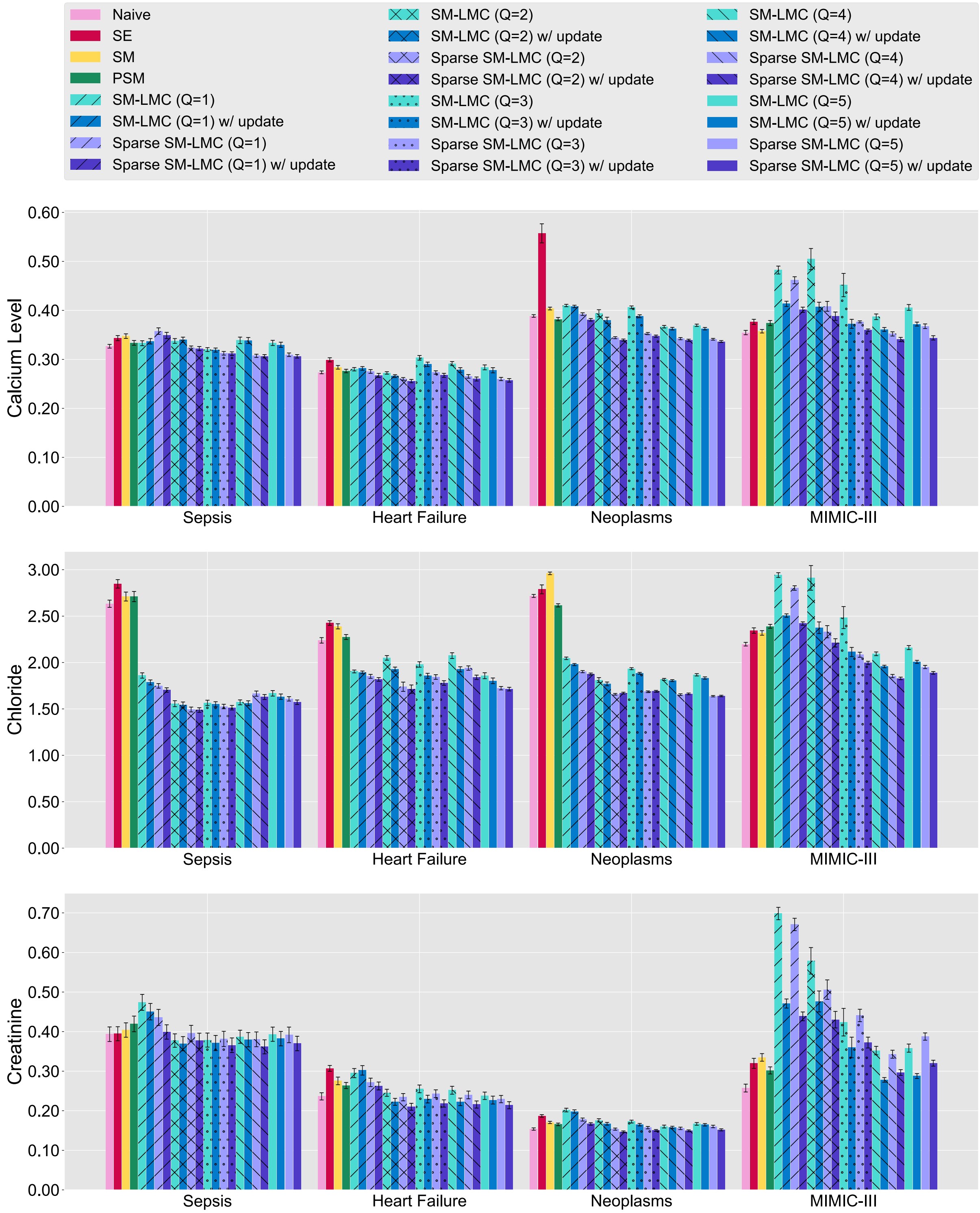

Figure H: The mean absolute error (MAE) of online imputation under different Q for all cohorts. The error bars denote ± 1 standard error.

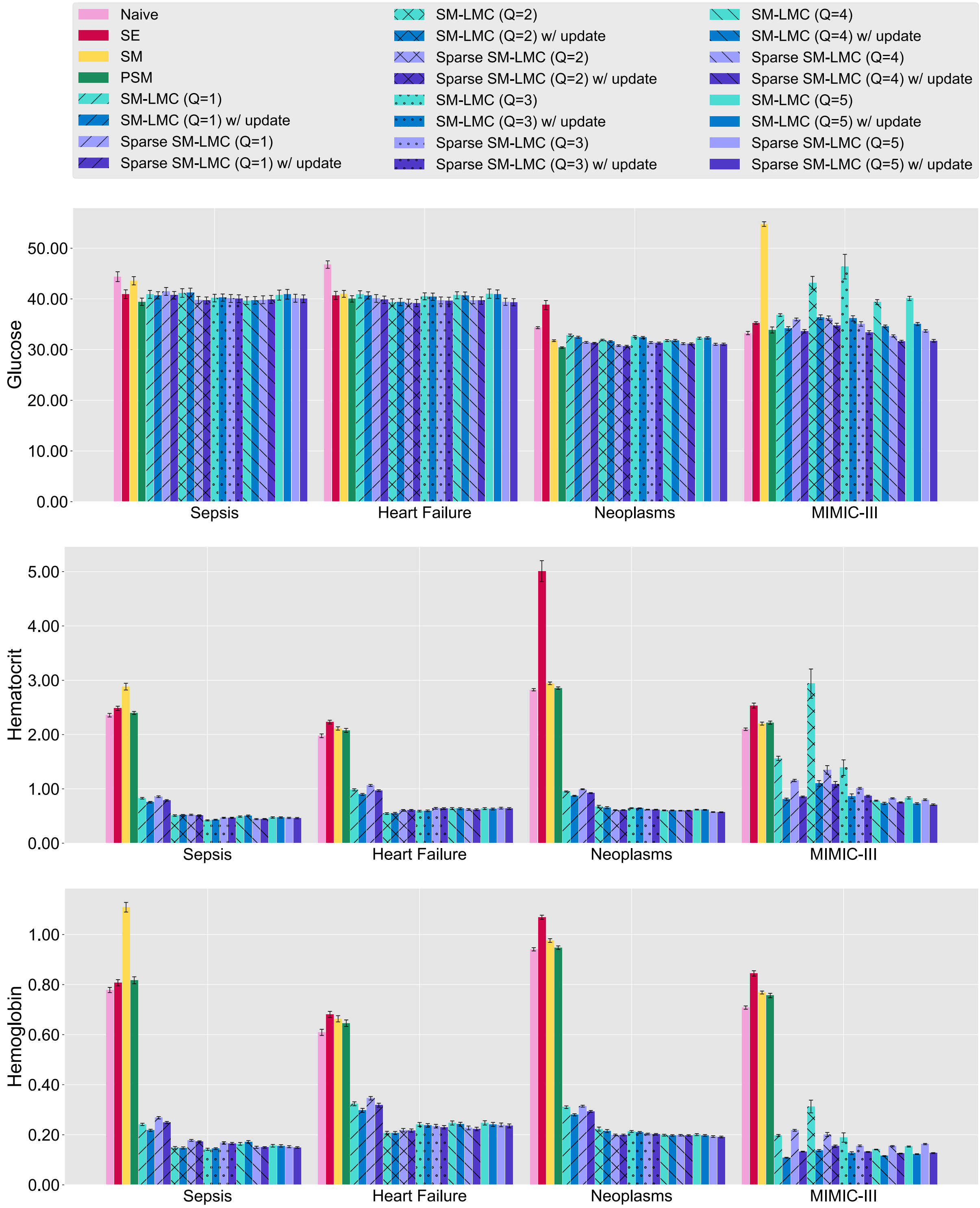

Figure I: The mean absolute error (MAE) of online imputation under different Q for all cohorts. The error bars denote ± 1 standard error.

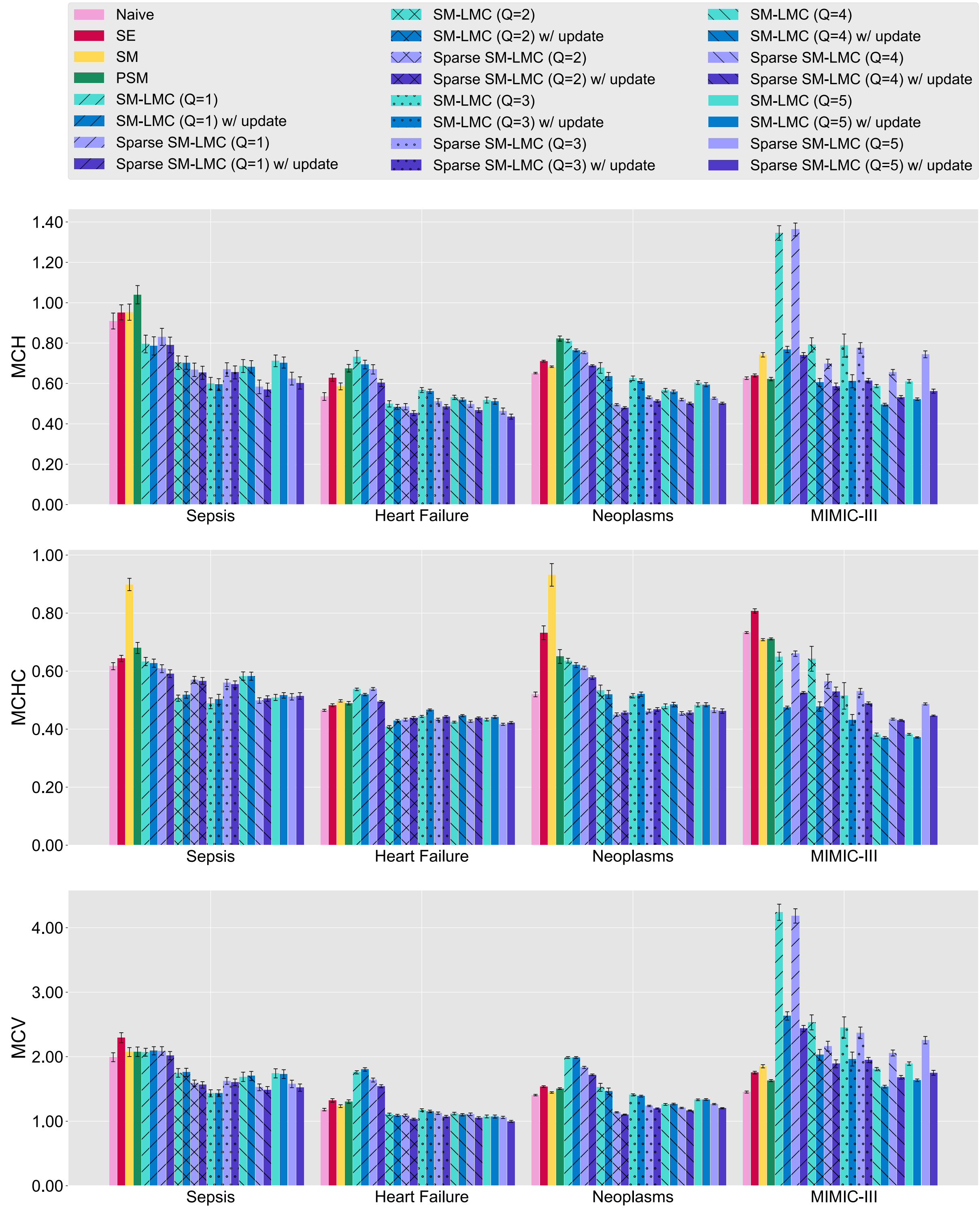

Figure J: The mean absolute error (MAE) of online imputation under different Q for all cohorts. The error bars denote ± 1 standard error.

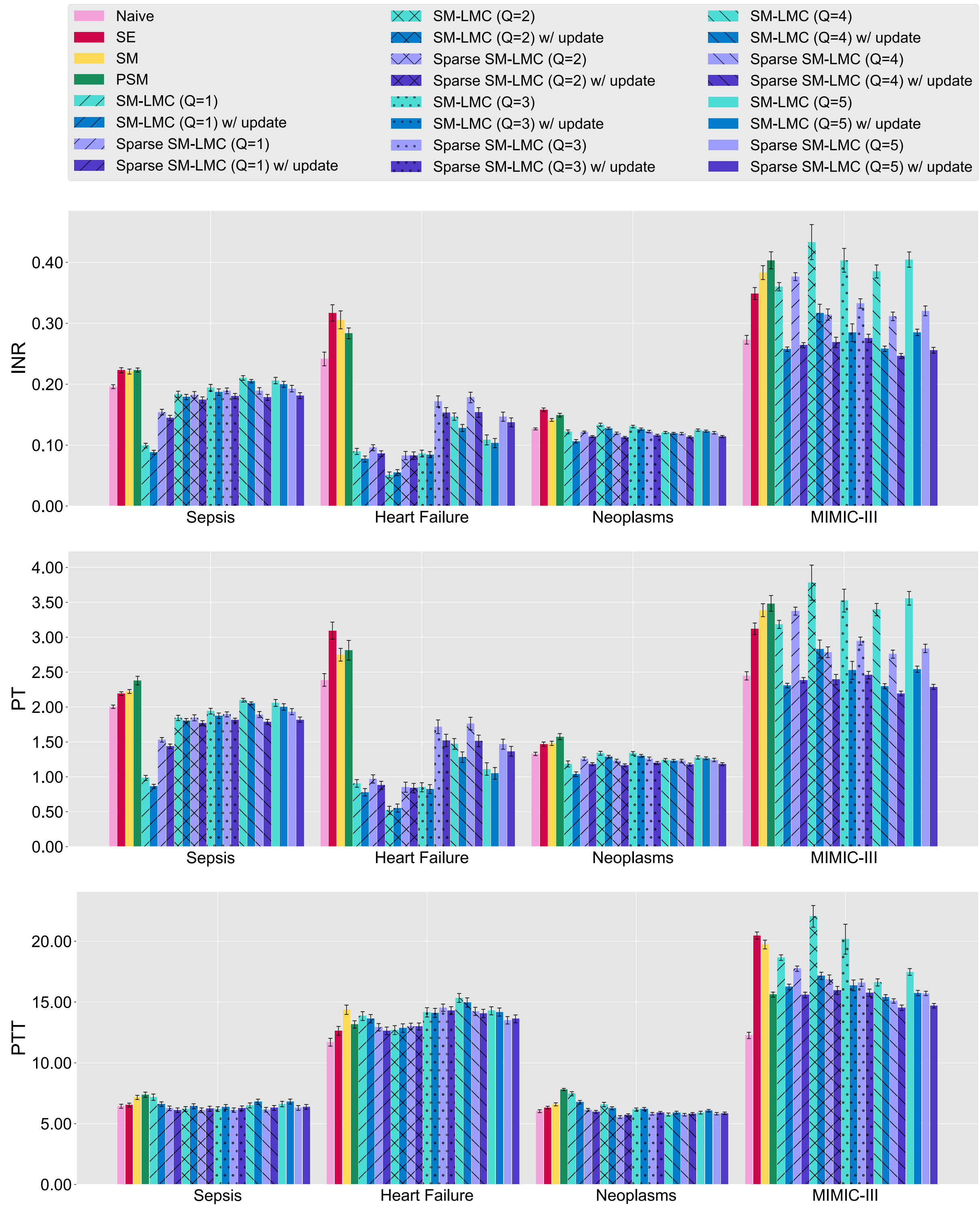

Figure K: The mean absolute error (MAE) of online imputation under different Q for all cohorts. The error bars denote ± 1 standard error.

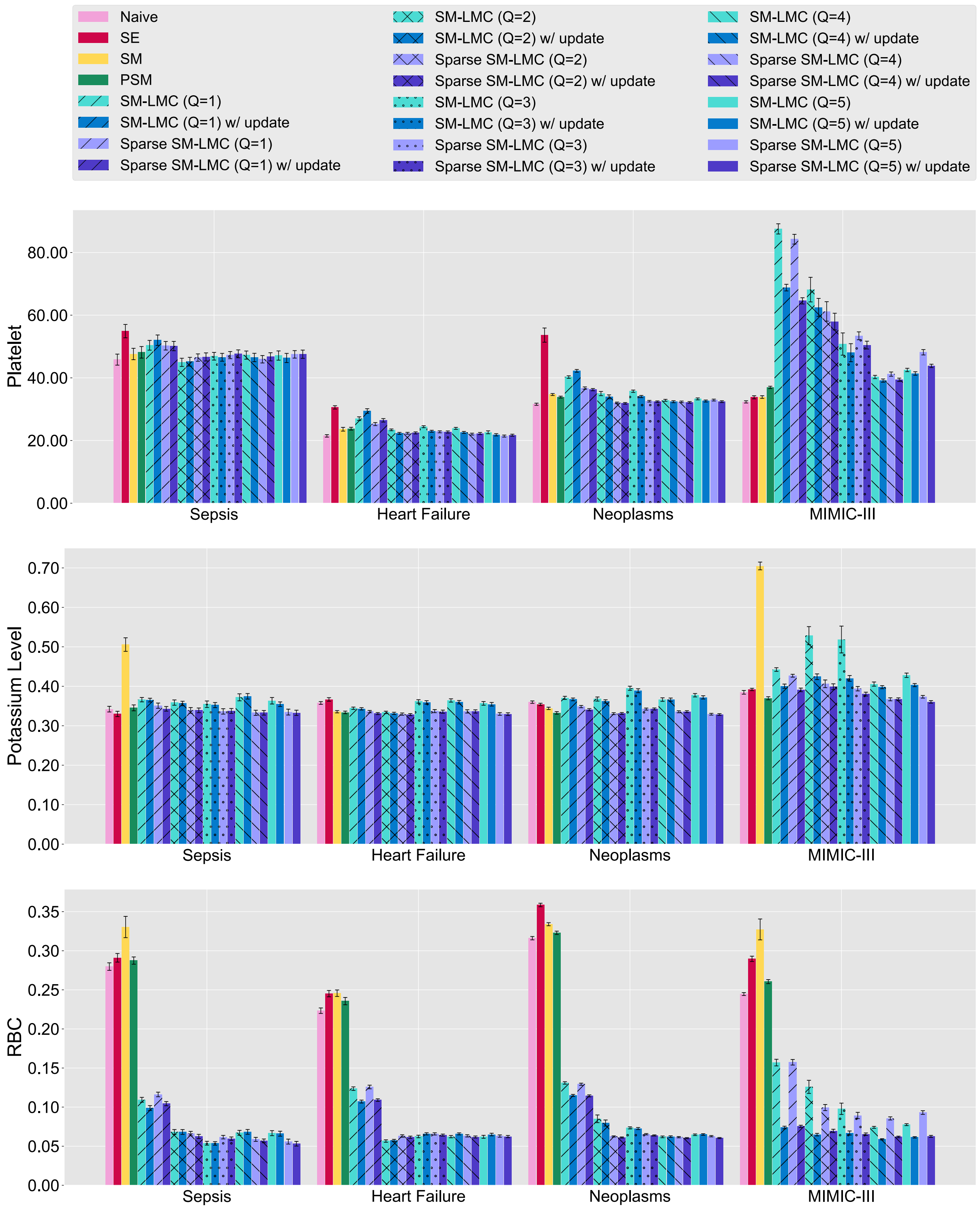

Figure L: The mean absolute error (MAE) of online imputation under different Q for all cohorts. The error bars denote ± 1 standard error.

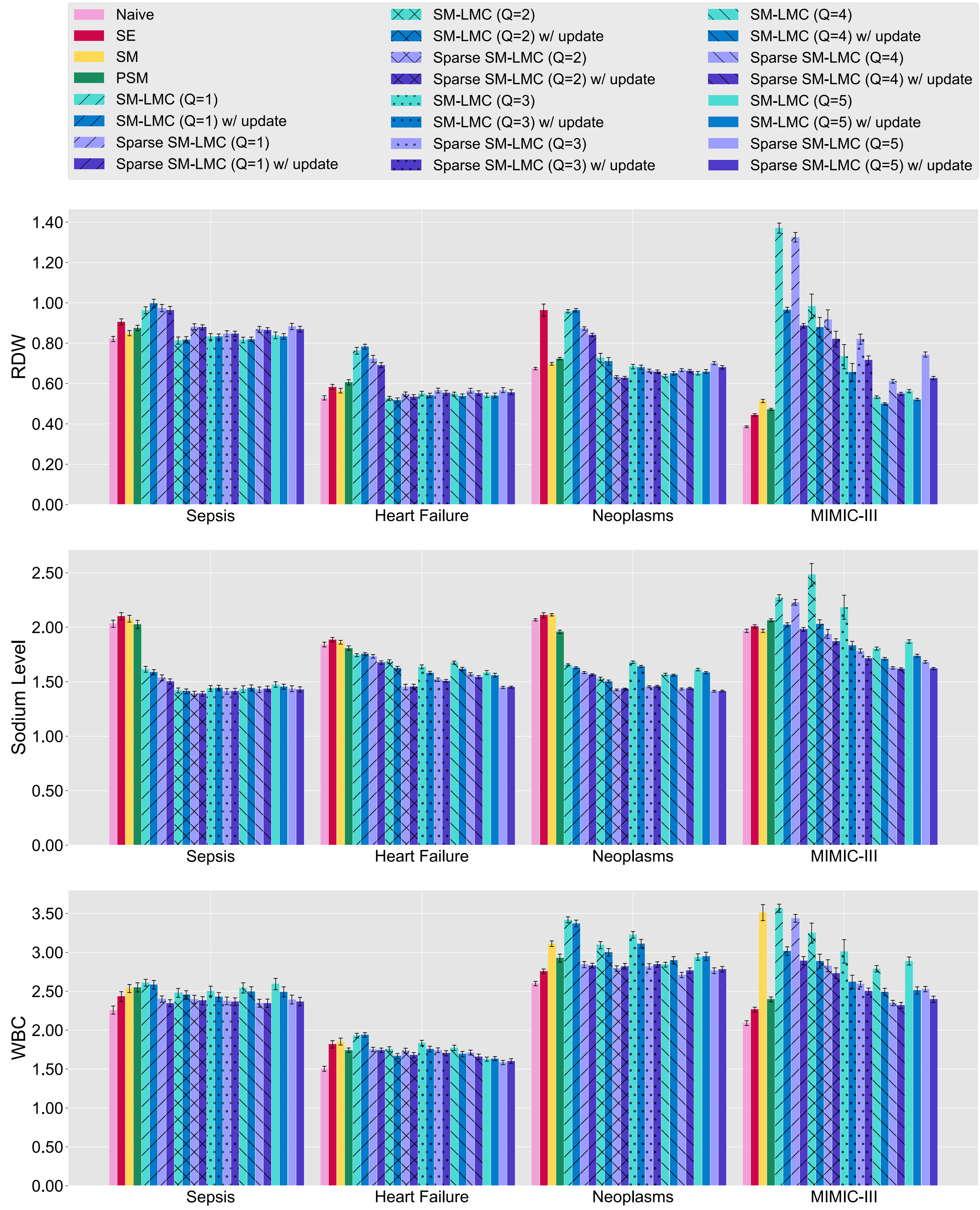

Figure M: The mean absolute error (MAE) of online imputation under different Q for all cohorts. The error bars denote ± 1 standard error.

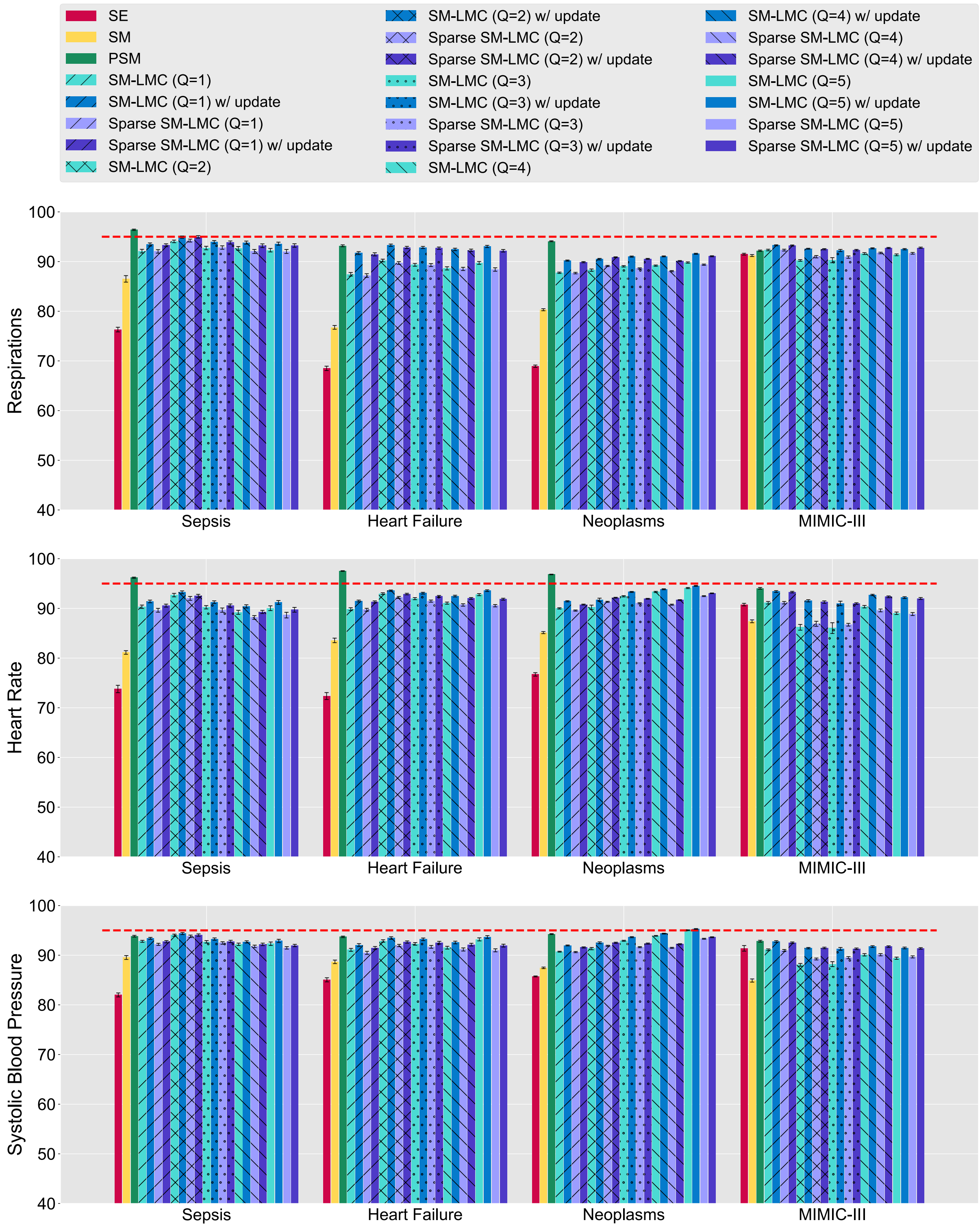

Figure N: The 95% coverage (in percentage) of online imputation under different Q for all cohorts. The error bars denote ± 1 standard error. The red dashed line indicates 95%.

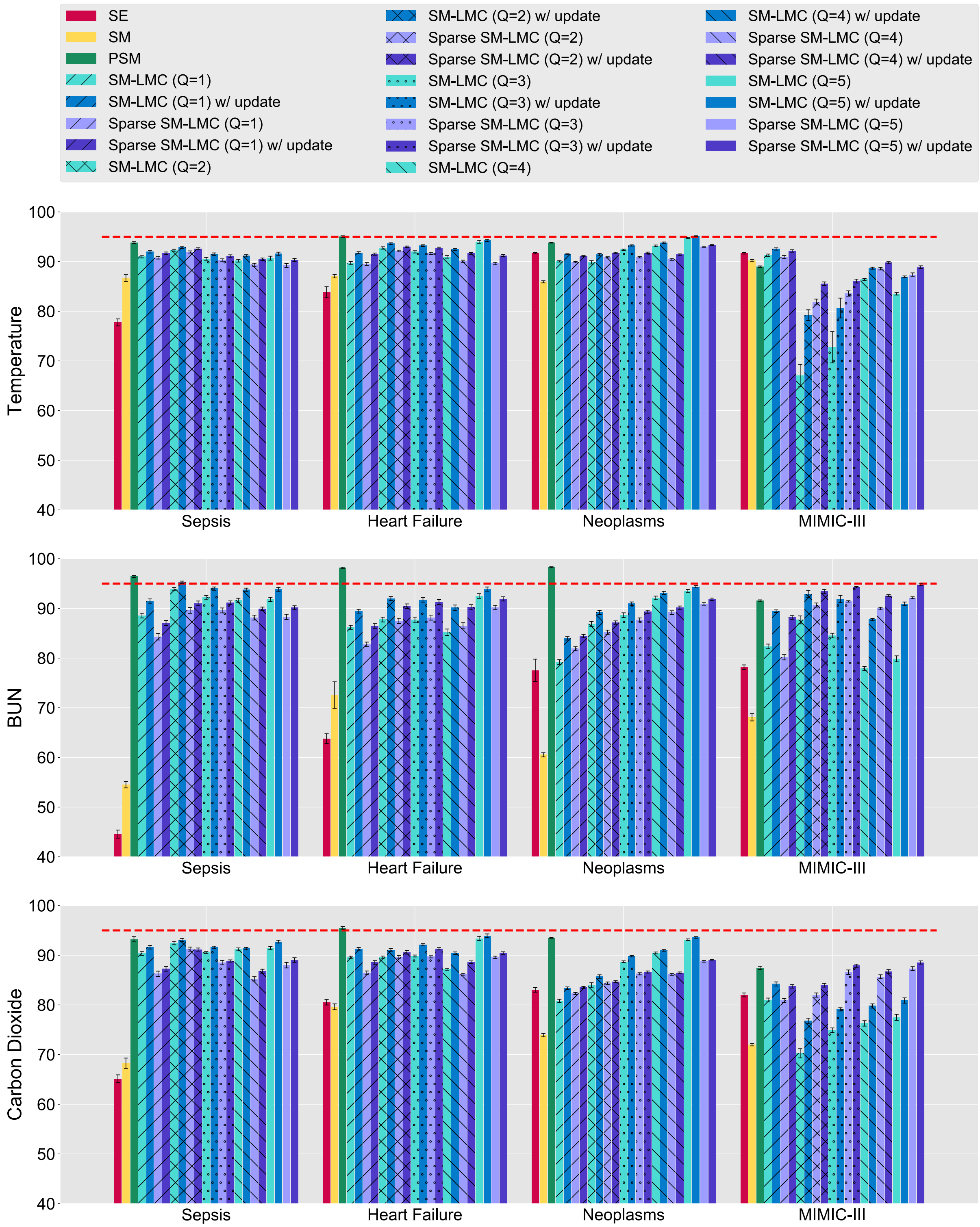

Figure O: The 95% coverage (in percentage) of online imputation under different Q for all cohorts. The error bars denote ± 1 standard error. The red dashed line indicates 95%.

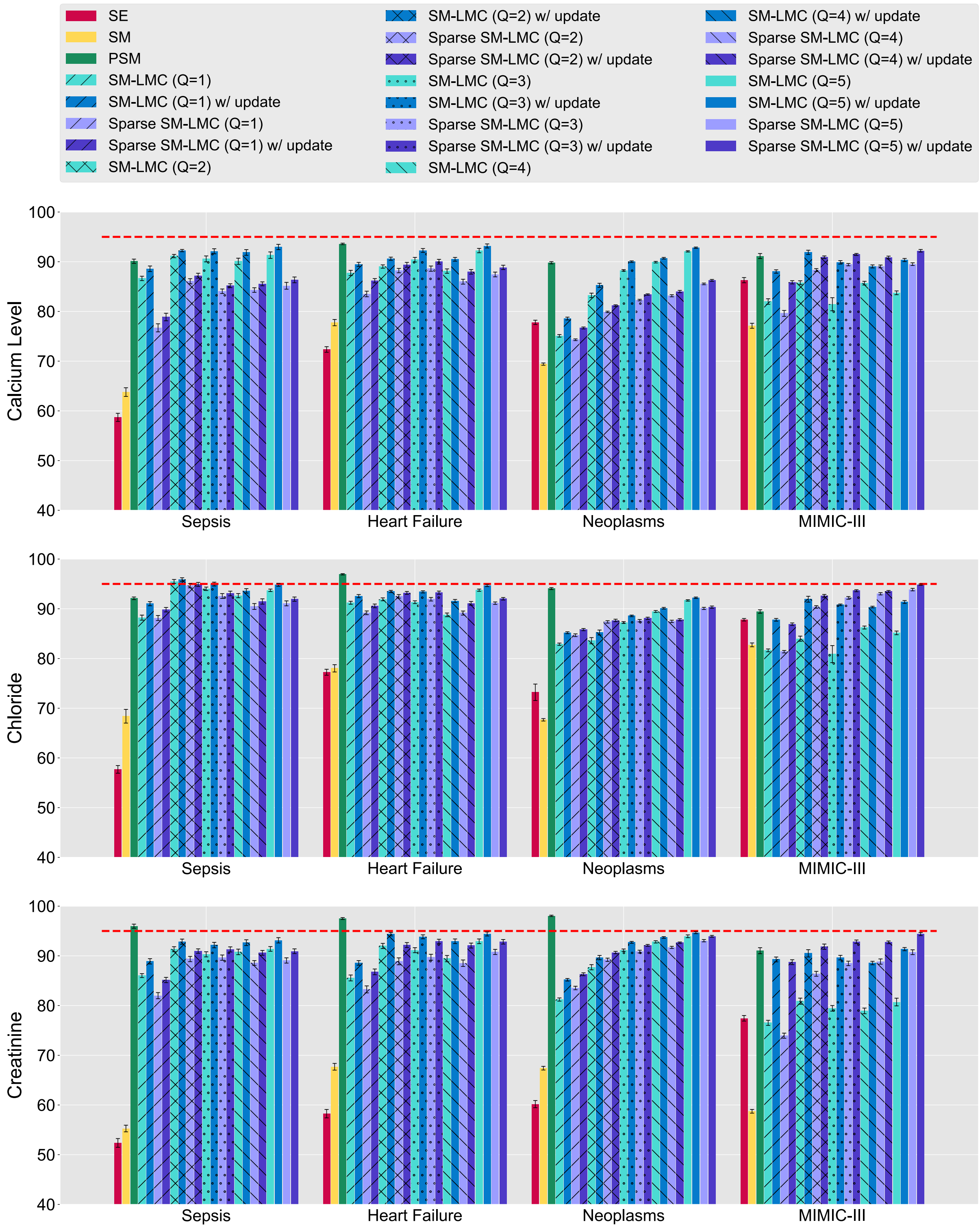

Figure P: The 95% coverage (in percentage) of online imputation under different Q for all cohorts. The error bars denote ± 1 standard error. The red dashed line indicates 95%.

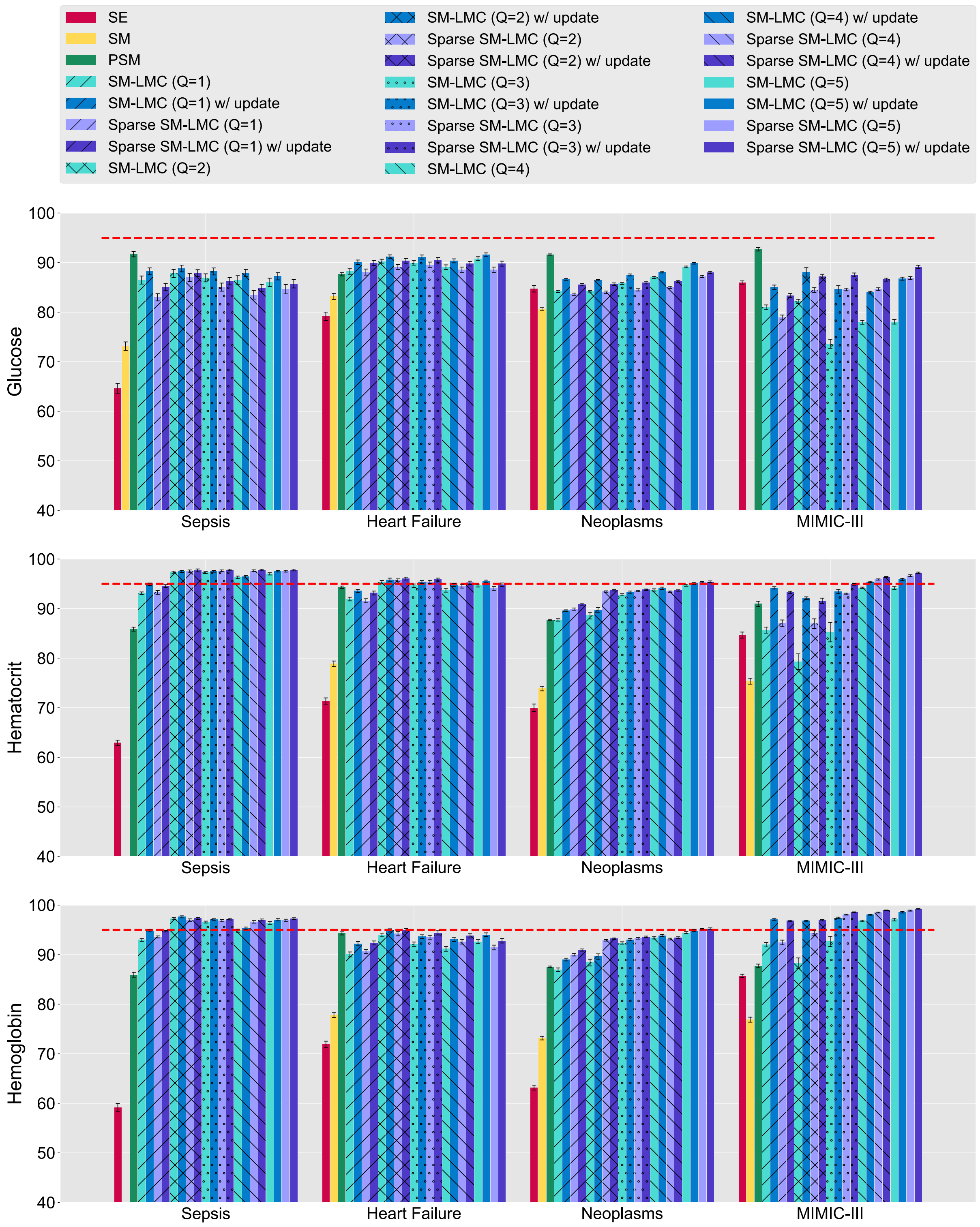

Figure Q: The 95% coverage (in percentage) of online imputation under different Q for all cohorts. The error bars denote ± 1 standard error. The red dashed line indicates 95%.

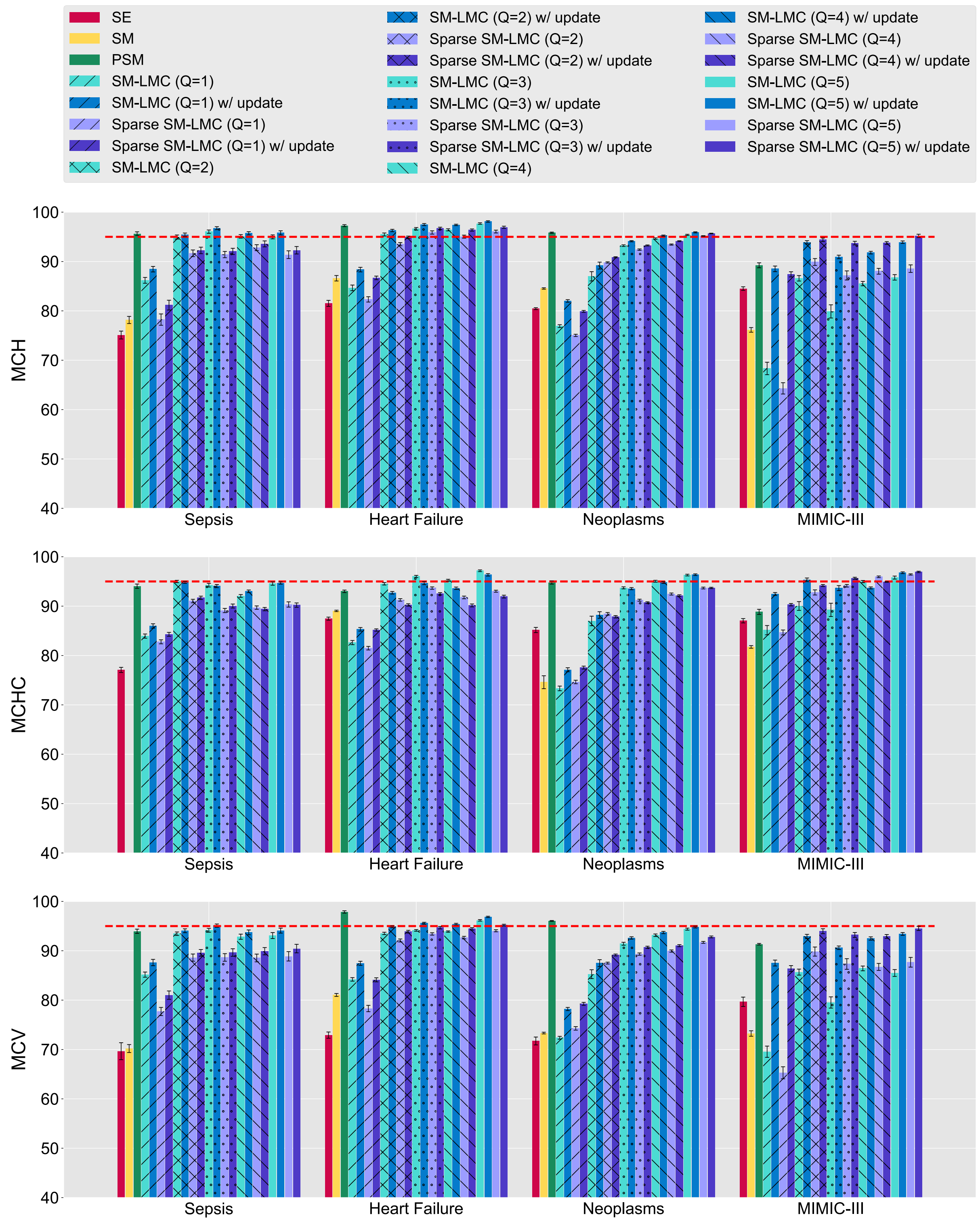

Figure R: The 95% coverage (in percentage) of online imputation under different Q for all cohorts. The error bars denote ± 1 standard error. The red dashed line indicates 95%.

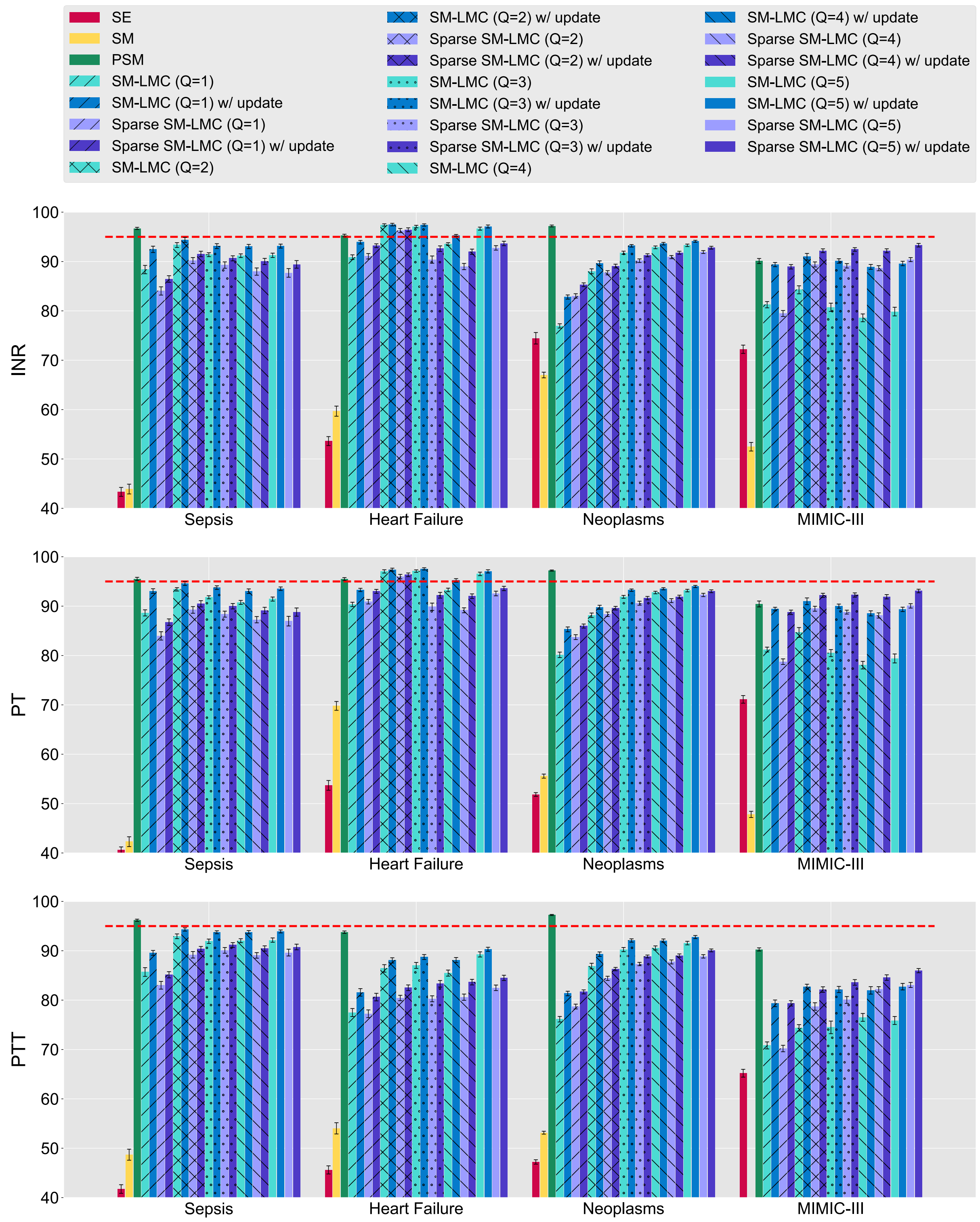

Figure S: The 95% coverage (in percentage) of online imputation under different Q for all cohorts. The error bars denote ± 1 standard error. The red dashed line indicates 95%.

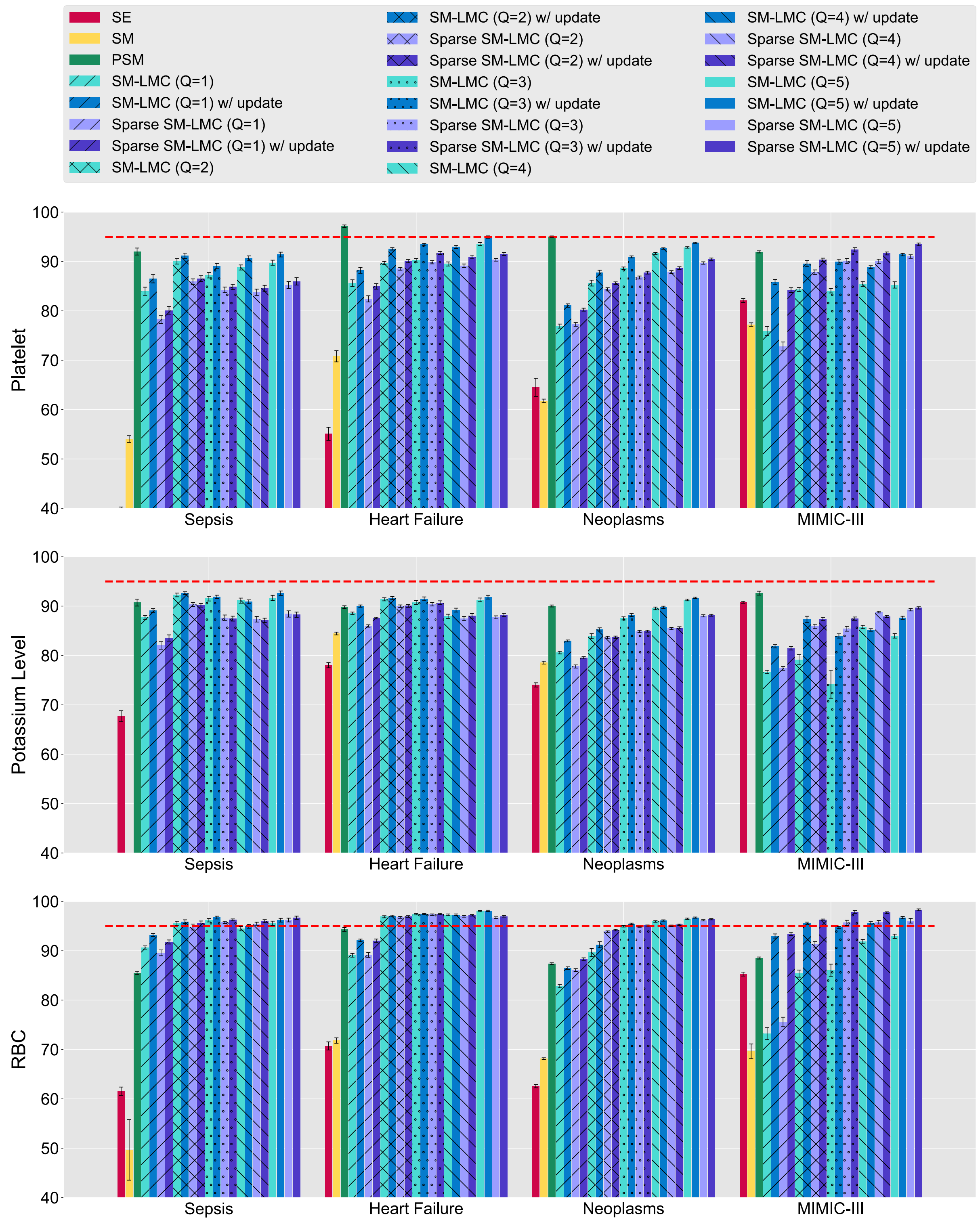

Figure T: The 95% coverage (in percentage) of online imputation under different Q for all cohorts. The error bars denote ±1 standard error. The red dashed line indicates 95%.

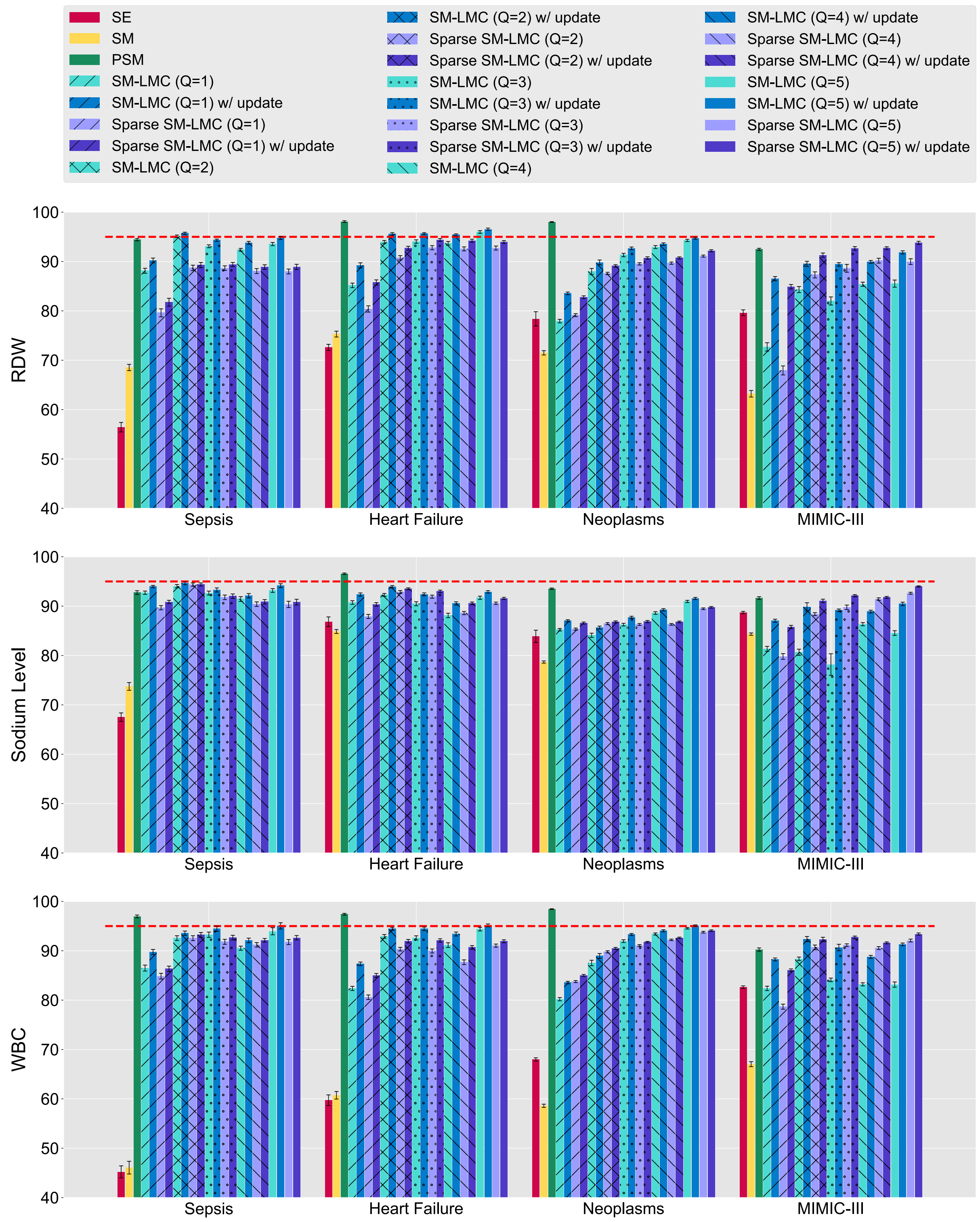

Figure U: The 95% coverage (in percentage) of online imputation under different Q for all cohorts. The error bars denote ± 1 standard error. The red dashed line indicates 95%.

Appendix E: Improvements in empirical runtime

In this appendix, we provide the comparisons in runtime with GPy (GPy, since 2012), a state-of-the-art optimized Python library for GPs. We selected few benchmark cases from the MIMIC-III subset, and profiled the runtime for performing one iteration when using gradient-based optimizers. That is, the runtime for computing the gram matrix, log marginal likelihood, and gradients of all parameters. The experiments were performed on the machine with 20 Intel(R) Xeon(R) CPUs running at 2.50GHz (no GPUs were used). For GPy implementation, we also allowed multithreading and the access to MKL optimization for matrix operations, provided by Anaconda with academic license. In Figure V, we show the average runtime for a single iteration under different number of basis kernels: $Q = 1$ and $Q = 5$, corresponding to 242 and 1114 parameters ($D = 24, R = 8$). We found that for training cases smaller than 10^4 observations, GPy with multithreading is comparable to our implementation. However, for the cases larger than 10^4 observations, our implementation speeds up by up to 2.5 times. The largest case we tested here includes 29,525 observations.

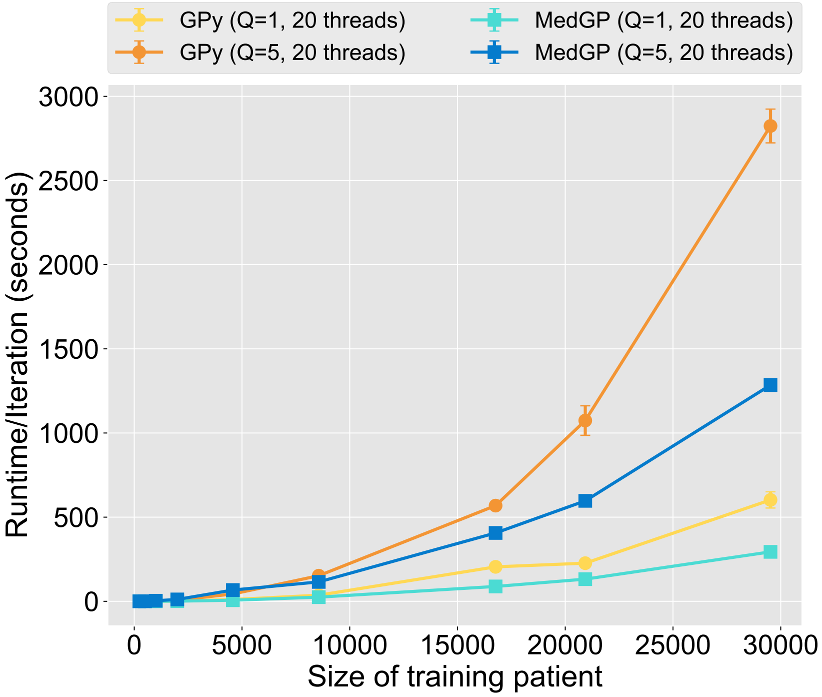

Figure V: The empirical runtime of our implementation. A comparison of the average runtimes for one iteration (including computation of gradients) for MedGP and optimized baseline GPy.

References

GPy. GPy: A gaussian process framework in python. `http://github.com/SheffieldML/GPy`, since 2012.
